# Intersecting evidence: Bibliometric analysis and clinical trials illuminate immunotherapy in KRAS-mutation cancer: A review

**DOI:** 10.1097/MD.0000000000039334

**Published:** 2024-09-06

**Authors:** Hongyang Liu, Min Qiang, Ying Zhang, Hong Wang, Yang Xing, Rui Guo

**Affiliations:** a The First Hospital of Jilin University, Chang Chun, China; b Cancer Center, The First Hospital of Jilin University, Chang Chun, China; c Clinical Laboratory, The First Hospital of Jilin University, Chang Chun, China.

**Keywords:** Citespace, clinical trials, immunotherapy, KRAS, scientometric analysis, targeted therapy, vaccine

## Abstract

KRAS mutations play a critical role in the development and progression of several cancers, including non-small cell lung cancer and pancreatic cancer. Despite advancements in targeted therapies, the management of KRAS-mutant tumors remains challenging. This study leverages bibliometric analysis and a comprehensive review of clinical trials to identify emerging immunotherapies and potential treatments for KRAS-related cancers. Using the Web of Science Core Collection and Citespace, we analyzed publications from January 2008 to March 2023 alongside 52 clinical trials from ClinicalTrials.gov and WHO’s registry, concentrating on immune checkpoint blockades (ICBs) and novel therapies. Our study highlights an increased focus on the tumor immune microenvironment and precision therapy. Clinical trials reveal the effectiveness of ICBs and the promising potential of T-cell receptor T-cell therapy and vaccines in treating KRAS-mutant cancers. ICBs, particularly in combination therapies, stand out in managing KRAS-mutant tumors. Identifying the tumor microenvironment and gene co-mutation profiles as key research areas, our findings advocate for multidisciplinary approaches to advance personalized cancer treatment. Future research should integrate genetic, immunological, and computational studies to unveil new therapeutic targets and refine treatment strategies for KRAS-mutant cancers.

## 1. Introduction

The KRAS oncogene stands as a pivotal node in the etiopathogenesis of various malignancies, harboring mutations in a significant proportion of cases. Specifically, KRAS mutations (KRAS MT) are implicated in an estimated 30% of lung adenocarcinomas, a staggering 45% of metastatic colorectal cancers, and an even more alarming 90% of pancreatic ductal adenocarcinomas.^[1–3]^ A noteworthy meta-analysis focusing on colorectal cancer illustrated a disconcerting picture, wherein progression-free survival (PFS) was notably compromised. The hazard ratio (HR) for PFS was quantified at 1.13, with a 95% confidence interval (CI) of 1.06 to 1.21, following first- or second-line systemic therapies that included agents such as irinotecan and oxaliplatin.^[4]^

Although recent strides in targeted therapy, most notably the advent of agents like Sotorasib targeting KRAS-G12C mutations, have achieved modest progress in enhancing antitumor efficacy, these advancements are not without limitations. Two seminal multicenter phase 1 to 2 trials involving Sotorasib revealed a median survival of 6.8 months (95% CI, 5.1–8.2) in KRAS-G12C lung cancer patients and 4.0 months (95% CI, 2.8–5.6) in those with pancreatic cancer.^[5,6]^ Ongoing trials for KRAS-G12D mutations further substantiate the burgeoning efforts in this arena (NCT05737706). However, it must be acknowledged that approximately one-quarter of patients exhibit adverse effects, which renders the search for alternative therapeutic strategies imperative. Intriguingly, there exists a mechanistic rationale suggesting that KRAS MT may induce immune evasion via PD-L1 upregulation through the MEK-ERK signaling pathway.^[7]^ This mechanistic insight lends credence to the exploration of immunotherapy as a viable treatment paradigm for KRAS MT tumors.

It is increasingly evident that the intricate molecular landscape of KRAS-mutant tumors harbors significant implications for therapeutic responsiveness. Clinical evidence corroborates the superiority of immunotherapy in prolonging overall survival (OS) relative to chemotherapy in patients with lung cancer (median OS of 4.6 [95% CI 2.8–6.7] months vs 4.2 months [95% CI 3.4–5.9]; *P* = .03).^[8]^ Further adding to the complexity of therapeutic outcomes are instances wherein KRAS is co-mutated with other genes, thus expanding the potential for nuanced immunotherapeutic strategies.

However, although immunotherapy has been shown to be superior, it cannot be used in all cases. Notably, the effectiveness of immune checkpoint blockades (ICBs) appears to be modulated by the presence of co-mutations. Patients with co-mutations involving TP53 and KRAS have shown augmented responses to immunotherapy, with a PFS of 33.3 months.^[9]^ This enhanced therapeutic response is posited to result from elevated PD-L1 expression following TP53 and KRAS co-mutations. On the contrary, co-mutations involving STK11/KEAP1 result in the diminished expression of PD-L1, thus compromising the therapeutic efficacy of ICBs.^[10]^ Recent clinical investigations substantiate this finding, as evidenced by shorter PFS and OS in individuals with STK11 and KRAS co-mutations.^[11]^

In the face of the complexity of immunotherapy applications to KRAS MT tumors, it is crucial to help new investigators quickly understand the research frontiers, developmental vectors, and hot directions of immunotherapy for KRAS MT tumors. Bibliometric analysis at this time is an invaluable tool for understanding temporal developments and emerging trends in KRAS-targeted immunotherapy.^[12,13]^ By comprehensively reviewing hundreds of scholarly papers, clinicians and researchers can gain a nuanced understanding of this rapidly evolving field, which can help to identify key therapeutic challenges and avenues for future research.

Therefore in this article, we use bibliometrics to meticulously analyze immunotherapy for KRAS MT tumors while incorporating exploratory clinical trials. We strive to clarify the developmental vein, find hot frontiers, predict future trends, and provide new insights and directions for future research.

## 2. Materials and methods

### 2.1. Scientometric analysis

**Figure 1.**
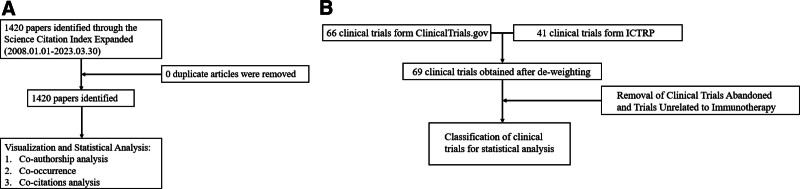
Flowchart of materials and methods.

Web of Science Core Collection (WoSCC) is the primary data source for Citespace (6.1.6) and VOS Browser (1.6.18), and the Extended Science Citation Index in WoSCC was used for this scientometric analysis.^[14]^ The search terms were the keywords related to immunotherapy from the Medical Subject Headings database AND KRAS. The citation space was adjusted according to the time slice (January 2008 to March 2023), the selection criteria (the first 50 layers of each slice), and the pruning function (to keep the default values). The study authors used the VOS viewer (1.6.18) for analysis. In addition to bibliometrics, we also analyzed clinical trials as corroboration as well as supplementation of the bibliometric content (Fig. 1A).

### 2.2. Clinical trials

To exhaustively investigate clinical trials related to KRAS and immunotherapy, we used 2 databases: ClinicalTrials.gov and the World Health Organization’s International Clinical Trials Registry Platform.^[15]^ As of March 2023, 66 clinical trials were initially retrieved from ClinicalTrials.gov and 41 additional trials were retrieved from International Clinical Trials Registry Platform. A total of 52 trials met the inclusion criteria, and we thoroughly analyzed the selected trials for various parameters including, but not limited to, treatment modality, targeted drug, treatment combination, trial phase, and current status (Fig. 1B).

## 3. Results

### 3.1. Scientometric analysis

#### 3.1.1. Distribution of publications and citations by year

Our examination of the scholarly landscape, encompassing 1420 peer-reviewed documents as cataloged by the WoSCC, yielded significant insights into the realm of immunotherapy for KRAS-induced malignancies. The median citation frequency per document was calculated to be 28.95, cumulatively amounting to a formidable 41,116 citations. Intriguingly, an H-index value of 95 was discerned, indicating that 95 seminal articles within this corpus have each been cited no fewer than 95 times, thereby serving as foundational pillars in the field.

**Figure 2.**
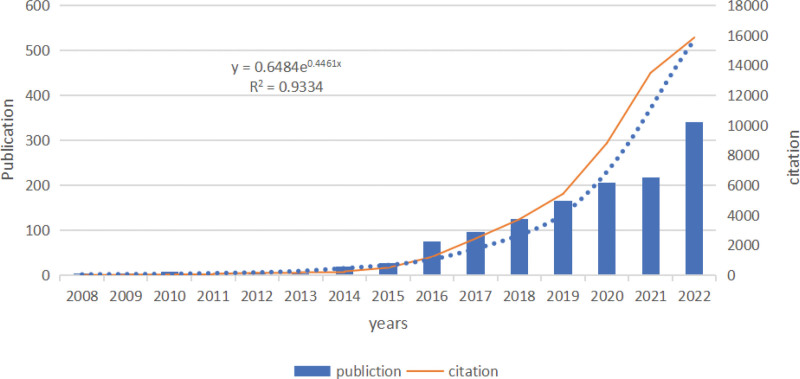
Distribution of publications and citations by year. The regression equation is *y* = 0.6484e^0.4461x^. Since the papers in this study do not cover all the papers in 2022, the expected number of papers in 2022 is calculated.

Temporal analysis of publication output and citations is graphically represented in Figure 2. The data spans from the year 2008 to 2023, highlighting an initial period of relative quiescence. Specifically, merely a solitary publication was disseminated in 2008, with a muted increase observed over the subsequent years until 2015. Nevertheless, the graph manifests an inflection point in 2016, after which a precipitous increase in academic contributions is evident.

To quantify these temporal variations and transitions, we employed an exponential regression model *y* = 0.6484*e*^0.4461*x*^. The model substantiates the observed trends, thus aiding in predictive analysis for future research activities in this continuously burgeoning field.

#### 3.1.2. Related countries (or regions) and institutions of this field

In the geographical analysis of 1420 academic papers, the United States demonstrated academic hegemony, publishing 623 papers, or about 43.8% of the total dataset. This was closely followed by China, which published 410 papers, accounting for approximately 28.9% of the research corpus.

**Figure 3.**
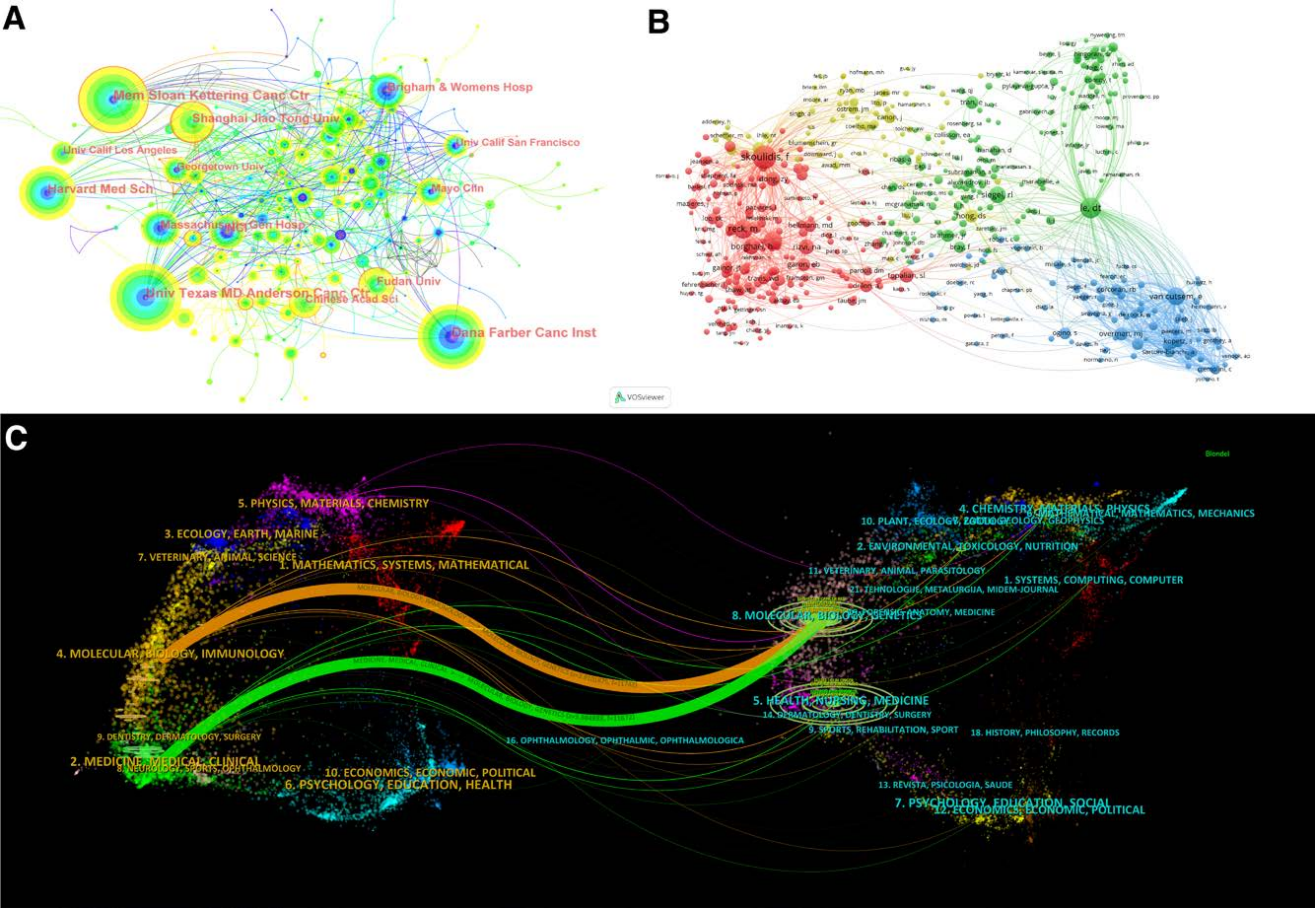
(A) Co-occurrence graph of research institutions. The size of the nodes depends on the number of papers published by the institution. The width of the circle around the node represents the degree of centrality between institutions. (B) Network graph of research authors with more than 40 co-citations. (C) A two-image overlay of journal interrelationships, where the line between them represents the citation trend.

The same trend was maintained in the institutional analysis. We used the centrality measure, and 6 institutions had centrality scores above 0.10. These included the University of Texas MD Anderson Cancer Center (centrality = 0.15), The Ohio State University (centrality = 0.11), the German Lung Research Center (centrality = 0.11), Shanghai Jiao Tong University (centrality = 0.10), the University of Pennsylvania (centrality = 0.10), and the National Cancer Institute (centrality = 0.10) , and the National Cancer Institute (centrality = 0.10). In addition, we conducted a network analysis to identify the institutional centers of gravity in this specialized domain (Fig. 3A). The study was then further narrowed down to focus on the author.

#### 3.1.3. Authors

The number of publications was first analyzed to determine the scientific output of each researcher, and 6 authors made substantial contributions, each publishing 10 or more articles in this area of specialization. The most important of these is G.K. Huang, who has authored 17 papers, thus becoming a key figure in the field.

However, there are limitations in judging by output alone. So we conducted a co-citation analysis. This analysis, which is often seen as a more nuanced measure of an author’s impact on a particular research topic, totaled 522 authors with at least 20 citations. The network of linked authors shown in Figure 3B helps to understand the dynamics of collaboration and influence in this specialized research area. Among the authors with a high number of co-citations, 18 authors have more than 100 citations. Of these, Skolidisf and LE DT are particularly influential, with the highest number of co-citations. Concluding the study from the direction of article output, we followed up by analyzing information about the journals as well as the articles themselves.

#### 3.1.4. Journal

As part of a meticulous scientometric analysis, we identified journals that serve as major platforms for disseminating groundbreaking research in the field of KRAS-induced cancer immunotherapy. According to our data, the top 5 journals in terms of citations were Journal of Clinical Oncology (4226 citations, impact factor 45.3), Clinical Cancer Research (3494 citations, impact factor 13.8), The New England Journal of Medicine (3577 citations, impact factor 176.1), Nature (2686 citations, impact factor 69.504), and Cancer Research (2297 citations, impact factor 11.2). These journals not only have strong impact factors, but also publish a large number of high-quality articles on KRAS-related tumors.

In order to decipher the complex interactions between these journals in a nuanced way, we used a two-image overlay generated via CiteSpace (Fig. 3C). This tool provides a two-dimensional graphical representation that illuminates the citation relationships between journals, thus providing valuable insights into the interdisciplinary nature of this study.

Specifically, our bi-graphic overlay shows 2 major citation paths, marked by different colored lines. The first citation path shows that articles published in molecular biology and immunology journals were primarily cited by articles in molecular biology and genetics journals. The second citation path shows a similar trend for studies published in medical and clinical journals, which are also frequently cited by articles in molecular biology and genetics journals.

#### 3.1.5. Reference

**Figure 4.**
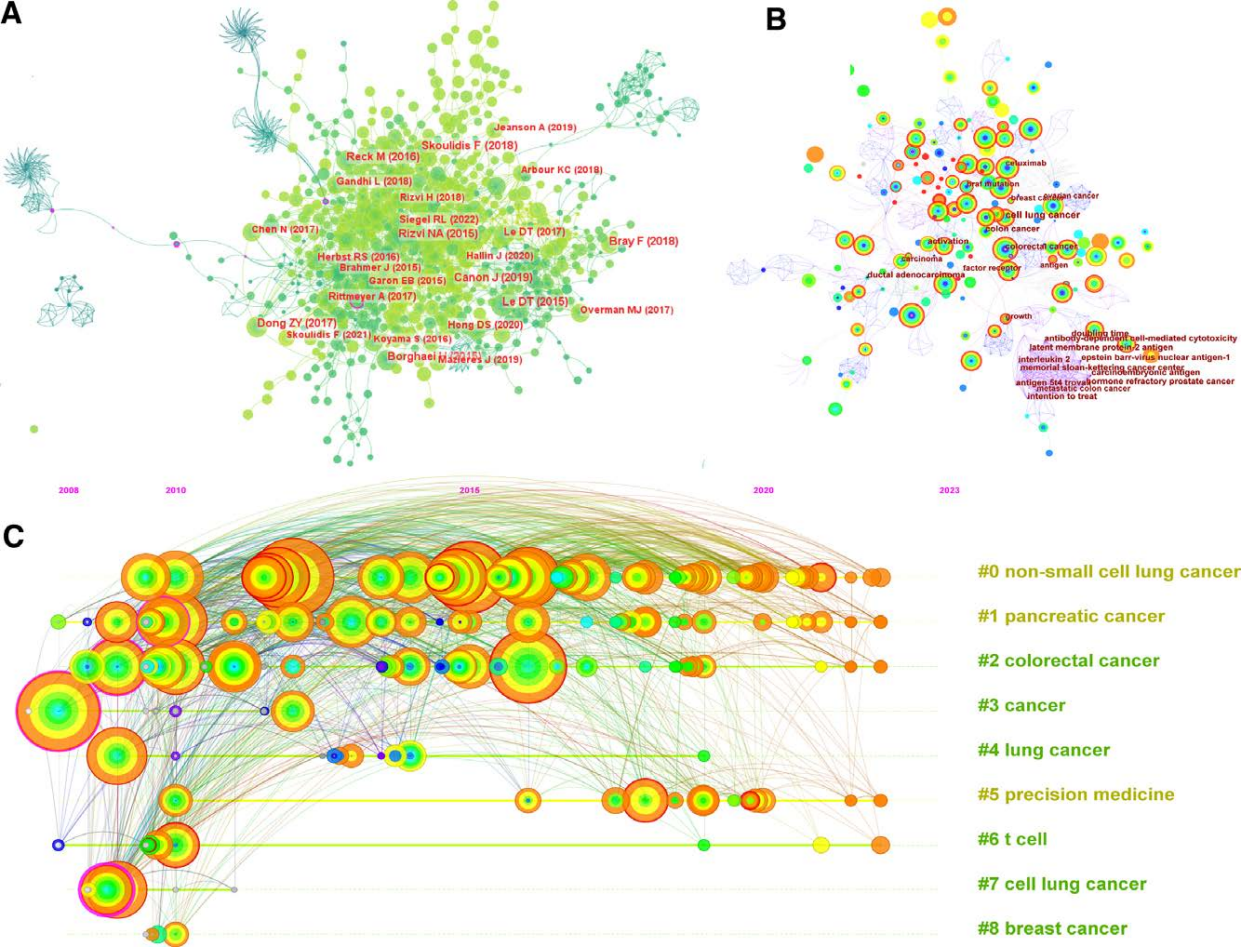
(A) Co-citation analysis of references. References with a high number of co-citations are organized by year. (B) Co-occurrence graph for high-frequency keywords. (C) Timeline view of keywords in Citespace. The top 10 clusters were selected for the analysis. The algorithm showing the cluster labels is LLR.

By performing a co-citation analysis of references through CiteSpace (Fig. 4A), we delineated the temporal landscape and evolving trends of research focused on KRAS-induced immunotherapy in cancer. The graph signifies that a majority of the highly-referenced papers were published within the last 5 years. This temporal concentration of research underscores the rapid advancements in this subject matter and the consequent need to remain contemporaneous with emergent data. We narrowed our scope further by selecting the top 10 highly-cited papers for analysis (Table 1).^[16–25]^ Among these, 9 are clinical trials that predominantly investigate different immune checkpoint inhibitors. Specifically, 5 trials are centered on PD-1 inhibitors, one on PD-L1, one on Nivolumab, one involves a KRAS-G12C inhibitor, and the remaining one on atezolizumab. After that, the refinement continues with a detailed analysis of the article’s keywords.

**Table 1:** The top 10 co-cited documents.

Year	Title	Type	First author	Journal	Focus and main idea	IF (2022)	JCR	Co-citation
2018	STK11/LKB1 mutations and PD-1 inhibitor resistance in KRAS-mutant lung adenocarcinoma	Basic Clinica1 Trials	Skoulidis F	Cancer Discovery	STK11/LKB1 mutations are the main cause of primary resistance to PD-1 inhibitors in KRAS-mutant lung cancer.	38.272	Q1	158
2016	Pembrolizumab versus chemotherapy for PD-L1-positive non-small-cell lung cancer	Basic Clinica1 Trials	Reck M	NEW ENGLAND JOURNAL OF MEDICINE	A Phase 3 trial shows pembrolizumab is better than platinum chemotherapy for advanced NSCLC with PD-L1 expression in 1%+ tumor cells.	176.082	Q1	126
2017	Potential predictive value of TP53 and KRAS mutation status for response to PD-1 blockade immunotherapy in lung adenocarcinoma	Basic Clinica1 Trials	Dong ZY	Clinical Cancer Research	TP53 and KRAS mutations in lung cancer may predict response to anti-PD-1/PD-L1 immunotherapy.	13.801	Q1	122
2015	Mutational landscape determines sensitivity to PD-1 blockade in non-small cell lung cancer	Basic Clinica1 Trials	Rizvi NA	Science	Whole-exome sequencing was done on NSCLC treated with pembrolizumab, an anti-PD-1 antibody.	63.832	Q1	116
2015	Nivolumab versus docetaxel in advanced nonsquamous non-small-cell lung cancer	Clinical Trial	Borghaei H	New England Journal of Medicine	A Phase III trial showed ibritumomab increased overall survival more than docetaxel in advanced non-squamous NSCLC patients who progressed after platinum chemotherapy.	176.082	Q1	116
2019	The clinical KRAS(G12C) inhibitor AMG 510 drives antitumor immunity	Clinical Trial	Canon J	Lancet	A Phase III trial found that bevacizumab with chemotherapy improved overall survival in advanced cervical cancer.	202.731	Q1	113
2018	Global cancer statistics 2018: GLOBOCAN estimates of incidence and mortality worldwide for 36 cancers in 185 countries	Review	Bray F	CA-Cancer J Clin	A status report on the global burden of cancer.	286.130	Q1	112
2015	PD-1 blockade in tumors with mismatch-repair deficiency	Clinical Trial	Le DT	New England Journal of Medicine	A study shows using Bloomberg-Mipidu to prevent immune checkpoints in a second phase experiment fixes previous errors and has clinical benefits.	176.082	Q1	108
2017	Atezolizumab versus docetaxel in patients with previously treated non-small-cell lung cancer (OAK): a phase 3, open-label, multicentre controlled trial	Clinical Trial	Rittmeyer A	Lancet	ATezolizumab improves NSCLC survival with good safety in a 3-stage study.	202.731	Q1	106
2017	Mismatch repair deficiency predicts the response of solid tumors to PD-1 blockade	Clinical Trial	Le DT	Science	Synthetic long-peptide vaccine effective in HPV-16-positive vulvar intraepithelial neoplasia.	63.832	Q1	102

#### 3.1.6. Co-occurrence analysis of keywords

We utilized CiteSpace to create a keyword co-occurrence graph (Fig. 4B), designed to unveil the relationship among terms and thereby identify the seminal content and emergent hotspots in the domain of KRAS-induced cancer immunotherapy. The graph pinpoints 6 keywords that recur with the highest frequency: “cancer” (n = 214), “expression” (n = 211), “mutation” (n = 175), “open-label” (n = 173), “immunotherapy” (n = 149), and “clathrate” (n = 133).

These recurring keywords not only reflect the primary research focus on KRAS mutations and immunotherapy in cancer but also provide insights into methodological aspects (“open-label”) and other molecular facets (“expression,” “clathrate”) that are pertinent to this line of inquiry.

#### 3.1.7. Timeline view of keywords

To provide a more nuanced understanding of the research domain, we clustered the keywords and generated a timeline analysis utilizing CiteSpace (Fig. 4C). This approach yielded 9 distinct clusters, each encapsulating a theme of importance in the context of KRAS-induced cancer immunotherapy. #0 Non-small cell lung cancer, #1 Pancreatic cancer, #2 Colorectal cancer, #3 Antigen, #4 Biliary tract cancer, #5 Treatment resistance, #6 Survival, #7 Lung cancer and #8 Lymph nodes. The first 3 clusters, corresponding to non-small cell lung cancer, pancreatic cancer, and colorectal cancer, indicate that the bulk of research involving KRAS mutations centers on these 3 tumor types.

#### 3.1.8. Burst analysis of keywords

**Figure 5.**
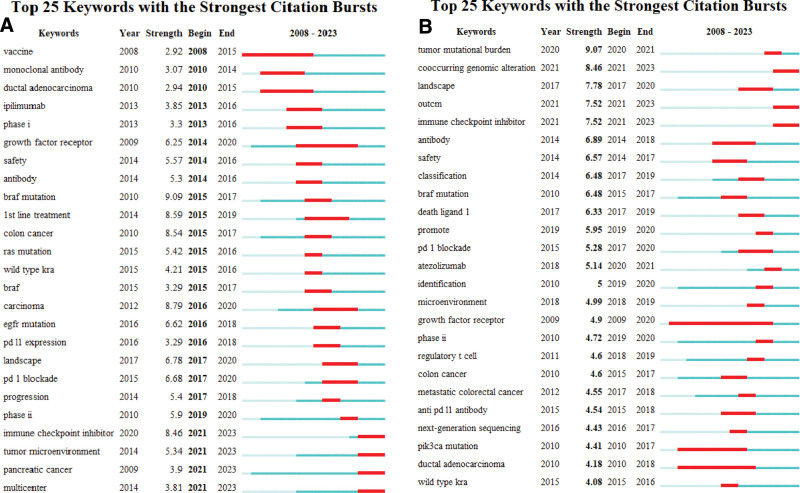
Top 25 most cited keywords in Citespace.

We employed burst detection techniques using Kleinberg algorithm, facilitated by CiteSpace, to identify keywords that have recently gained heightened attention in the research community. This algorithmic approach is particularly advantageous for recognizing emergent themes or “bursts” that signal the rise of new scientific paradigms or shifts in research focus.^[26]^ Among the 35 scrutinized keywords, we prioritized 25 based on their burst strength and temporal relevance (Fig. 5A and B). The keyword with the most potent burst was “BRAF mutation,” which had a burst strength of 6.41. It gained prominence in the year 2010 and maintained its relevance for a subsequent seven-year period. Further categorization of these burst keywords facilitated the demarcation of 2 primary thematic areas: (1) cancers induced by KRAS mutations and (2) approaches to cancer treatment, with a predominance of immunotherapy-related terms.

In addition to keywords that have been around for a long time, there have been many new keywords that have come up enthusiastically in recent years. Recent “buzzwords” like “tumor-infiltrating lymphocytes,” “checkpoint blockade,” and “PD-1 blockade” suggest that there is a focused shift towards understanding and enhancing the immunotherapeutic approaches for treating KRAS-induced malignancies.

### 3.2. Clinical trials

#### 3.2.1. Development and current status of related clinical trials

**Figure 6.**
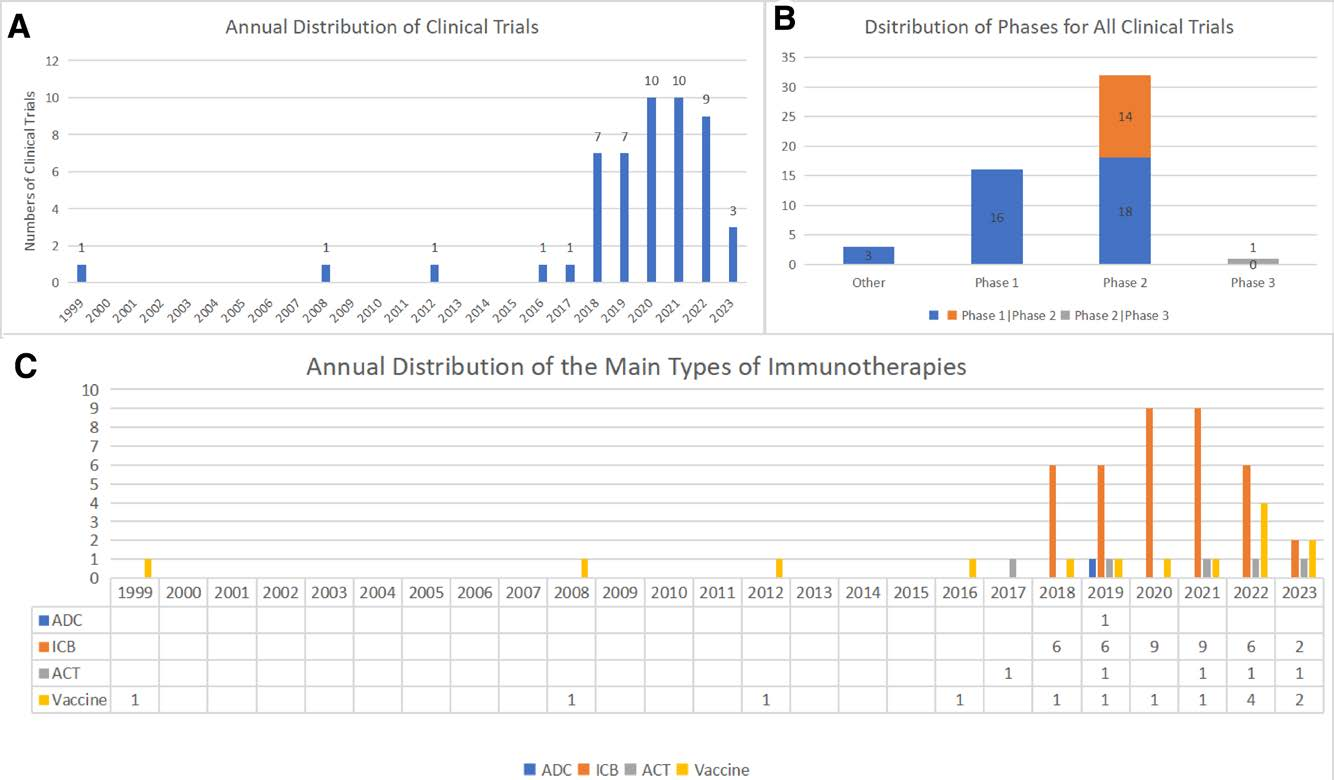
(A) Annual distribution of clinical trials. The start date is considered as the time of the clinical trial. (B) Phase distribution of all clinical trials. Other refers to trials with no known phase. (C) Yearly distribution of ICBs, ACTs, and vaccines, where the start date is considered as the time of the clinical trial. ACT = adoptive cell transfer therapy, ICB = immune checkpoint blockade.

The first vaccine therapy and Sargramostim trial for non-small cell lung cancer patients was conducted in 1999, marking the beginning of immunotherapy for KRAS malignancies (NCT00005630). Since then, there have been significant advancements in immunotherapy for KRAS-driven malignancies. Immune checkpoint blockers have shown impressive efficacy against these cancers, leading to a surge in clinical trials in recent years (Fig. 6A). And the phases of clinical trials (Fig. 6B) were mainly Phase II and Phase I. Clinical trials reached a peak in 2020 and 2021, with 10 trials conducted. It is anticipated that clinical trials will continue to increase in the future.

#### 3.2.2. Immunotherapeutic approaches used in relevant clinical studies

The most commonly used immunotherapies for treating KRAS-related cancers are ICB, vaccination, and adoptive cell transfer (ACT). Based on recent clinical trials and yearly distribution (Fig. 6C), ICB was the most studied immunotherapy (n = 39), followed by vaccination therapy. Vaccine therapy has a long history, with its first clinical trial conducted in 1999, and has gained sustained attention. Clinical trials related to vaccination therapy have increased in recent years, highlighting its potential in treating KRAS-induced malignancies (Table 2).

**Table 2:** Vaccine therapy.

No.	Trial ID	ICB	Vaccine	Phase	Start date	Title
1	NCT03948763	Pembrolizumab	V941	Phase 1	June 26, 2019	A Study of mRNA-5671/V941 as Monotherapy and in Combination With Pembrolizumab (V941-001)
2	NCT04117087	Ipilimumab/Nivolumab	KRAS peptide vaccine	Phase 1	May 29, 2020	Pooled Mutant KRAS-Targeted Long Peptide Vaccine Combined With Nivolumab and Ipilimumab for Patients With Resected MMR-p Colorectal and Pancreatic Cancer
3	NCT05631899	Anti-PD-1 antibody	KRAS-EphA-2-CAR-DC	Phase 1	February 3, 2023	Combination of CAR-DC Vaccine and Anti-PD-1 Antibody in Local Advanced/Metastatic Solid Tumors
4	NCT02380443		AlloStim	Phase 2	September 1, 2016	AlloStim Immunotherapy Dosing Alone or in Combination With Cryoablation in Metastatic Colorectal Cancer
5	NCT03592888		mDC3/8-KRAS Vaccine	Phase 1	November 20, 2018	DC Vaccine in Pancreatic Cancer
6	NCT04853017		ELI-002 7P	Phase 1	October 4, 2021	A Study of ELI-002 in Subjects With KRAS Mutated Pancreatic Ductal Adenocarcinoma (PDAC) and Other Solid Tumors

CAR-DC = chimeric antigen receptor dendritic cell, ICB = immune checkpoint blockade.

#### 3.2.3. Clinical trials of some important immunotherapeutic agents

Most newly developed immunotherapeutics for treating KRAS-related cancers are ICBs, with PD-1 and PD-L1 being prime examples. There are 12 vaccine-related trials, with 5 involving vaccination and ICB treatment. Four trials involve ACT, all of which are T-cell receptor T-cell (TCR-T). Targeted therapy and chemotherapy combined with immunotherapy are used more often in treating KRAS-induced cancers. The largest number of combinations involves targeted therapy and ICB, with 21 trials (Table 3). Other combinations include chemotherapy with ICB (3 trials), cell infusion with ICB (1 trial), and chimeric antigen receptor dendritic cell with ICB (1 trial). Six trials involve chemotherapy with targeted therapy and ICB (Table 4). The largest number of targeted combination ICB trials occurred in 2020 and 2021, with 9 and 6 trials, respectively.

**Table 3:** ICB combined with targeted therapy.

No	Trial ID	Targeted drugs	ICB	Phase	Start date	Title
1	ISRCTN13464104	MRTX849	Pembrolizumab	Phase II	November 23, 2020	A phase II trial of MRTX849 monotherapy and in combination with pembrolizumab in patients with advanced non-small-cell lung cancer with KRAS G12C mutation
2	NCT04613596	MRTX849	Pembrolizumab	Phase 2 Phase 3	December 2, 2020	Phase 2 trial of MRTX849 monotherapy and in combination with pembrolizumab and a phase 3 trial of adagrasib in combination in patients KRAS G12C mutation KRYSTAL-7
3	NCT04699188	JDQ443/ TNO155	Tislelizumab	Phase 1 Phase 2	February 24, 2021	Study of JDQ443 in patients with advanced solid tumors harboring the KRAS G12C mutation
4	NCT04745130	Regofinib/cetuximab/ regafinil	Sintilimab	Phase 2	March 1, 2021	Efficacy and safety of sintilimab combined with regorafenib and cetuximab/ sintilimab combined with regorafenib in posterior line therapy of advanced colorectal cancer (Regosinti)
5	EUCTR2020-003101-58-IT	MRTX849	Pembrolizumab	Phase 2	March 2, 2021	A phase 2 trial of MRTX849 in Combination with pembrolizumab in patients with advanced non-small cell lung cancer with KRAS G12C mutation—na
6	NCT04720976	JAB-3312/Binimetinib/ Sotorasib/Osimertinib	Pembrolizumab	Phase 1 Phase 2	March 23, 2021	JAB-3312 based combination therapy in adult patients with advanced solid tumors
7	EUCTR2020-003101-58-HU	MRTX849	Pembrolizumab	Phase 2	April 8, 2022	A phase 2 trial of MRTX849 monotherapy and in combination with pembrolizumab in patients with advanced non-small cell lung cancer with KRAS G12C mutation
8	NCT05609578	Adagrasib	Pembrolizumab	Phase 2	July 29, 2022	Combination therapies with adagrasib in patients with advanced NSCLC with KRAS G12C mutation
9	NCT05375084	BBP-398	Nivolumab	Phase 1	October 20, 2022	SHP2 inhibitor BBP-398 in combination with nivolumab in patients with advanced non-small cell lung cancer with a KRAS mutation
10	NCT05472623	Adagrasib	Adagrasib/Nivolumab	Phase 2	March 21, 2023	Neoadjuvant KRAS G12C directed therapy with adagrasib (MRTX849) with or without nivolumab

ICB = immune checkpoint blockade.

**Table 4:** Chemotherapy combined with targeted combined with ICB.

No.	Trial ID	Targeted drugs	ICB	Chemotherapy drugs	Phase	Title
1	NCT03777124	Apatinib	SHR-1210	Carboplatin/pemetrexed	Phase 2	Phase II study of SHR-1210 (anti-PD-1 antibody) combination with apatinib versus pemetrexed and carboplatin in subjects with KRAS mutant stage IV non-squamous non-small cell lung cancer
2	NCT04185883	Sotorasib/AMG 404/ trametinib/RMC-4630/ afatinib/BI 1701963	Pembrolizumab	Carboplatin/ pemetrexed/ docetaxel/ paclitaxel	Phase 1/Phase 2	A phase 1b/2, protocol evaluating the safety, tolerability, pharmacokinetics, and efficacy of sotorasib monotherapy and in combination with other anticancer therapies in subjects with advanced solid tumors with KRAS p.G12C mutation (CodeBreak 101)
3	NCT04214418	Cobimetinib	Atezolizumab	Hydroxychloroquine	Phase 1 Phase 2	Study of combination therapy with the MEK inhibitor, cobimetinib, immune checkpoint blockade, atezolizumab, and the AUTOphagy inhibitor, hydroxychloroquine in KRAS-mutated advanced malignancies
4	NCT04348045	Olaparib/selumetinib	Durvalumab	ARM C FOLFIRI	Phase 2	MAZEPPA: phase II PRODIGE-GERCOR study to evaluate MAintenance therapy with olaparib or selumetinib plus durvalumab according to BRCAness and KRAS somatic status personalized in metastatic pancreatic adenocarcinoma patients
5	NCT04956640	LY3537982/Abemaciclib/LY3295668/Cetuximab	Pembrolizumab	Pemetrexed/Cisplatin/Carboplatin	Phase 1	A phase 1a/1b study of LY3537982 in patients with KRAS G12C-mutant advanced solid tumors
6	NCT05504278	Cetuximab/IBI351	Sintilimab	Pemetrexed/cis-platinum/carboplatin	Phase 1	Efficacy and safety of IBI351 in combination with sintilimab chemotherapy in advanced non-squamous non-small cell lung cancer subjects with KRAS G12C mutation

ICB = immune checkpoint blockade.

## 4. Discussion

### 4.1. Analysis of bibliometric results

Bibliometric analysis serves as a pivotal exploratory tool, designed to dissect and elegantly delineate the intricate web of knowledge relationships within academic research.^[27]^ This method not only unpacks the structural dynamics and evolutionary trajectory of scientific inquiry but also illuminates the sprawling network of intellectual discourse. Our analysis of publication trends underscores a burgeoning interest in the field, with the United States and China emerging as leading contributors. This surge underscores the global scientific community’s growing engagement with this domain.

Within the corpus of our study, we have spotlighted ten seminal works that stand as cornerstones for researchers embarking on their journey into this field. These pivotal articles, alongside a curated selection of high-impact journals, constitute invaluable resources for those new to the discipline, offering a gateway to the wealth of knowledge and ongoing discourse.

Further, through meticulous keyword analysis, we distilled the discourse into 2 critical themes: gene co-mutation expression profiles and the tumor microenvironment. These themes not only encapsulate key areas of current research focus but also signal the rich potential for future exploration. Below, we delve deeper into these themes, unpacking their significance and the implications for advancing the frontier of scientific understanding in this realm.

#### 4.1.1. Gene co-mutation expression profiles

This key word underscored the clinical imperative for gene expression profiling in KRAS-mutated neoplasms, as these mutations frequently co-occur with additional genomic alterations. Such profiling is quintessential for gauging the efficacy and tolerability of immunotherapeutic agents, thereby accentuating the importance of personalized treatment regimens. Furthermore, a preponderance of patients with KRAS mutations manifest elevated levels of tumor-infiltrating lymphocytes.^[28]^ As such, inhibitors of the PD-1 and PD-L1 pathways are increasingly recognized as pivotal components of therapeutic strategies for KRAS-mediated malignancies.^[29,30]^ However, heterogeneity in treatment response exists; some patients with elevated PD-L1 expression and dense lymphocytic infiltration may not benefit from anti-PD-1 or anti-PD-L1 therapies.^[31,32]^ Pertinently, KRAS-STK11 or KEAP1 co-mutant lung adenocarcinomas present unique challenges, often characterized by attenuated immune markers, scant tumor-infiltrating lymphocytes, and subdued PD-L1 expression.^[33]^ Moreover, mutations in the STK11 gene can incite a phenotypic transition in lung adenocarcinoma to squamous cell carcinoma, a more aggressive and less prognostically favorable variant.^[34]^ Subtypes such as KRAS(MT) + STK11/LKB1(MT) and KRAS(MT) + STK11/KEAP1(MT) correlate with inferior progression-free and OS metrics compared to their wild-type counterparts.^[10,16,35]^ Despite the general ineffectiveness of immunotherapy against tumors with concurrent KRAS and STK11 mutations, isolated case reports suggest potential responsiveness to combinatorial regimens involving carboplatin, pemetrexed, and pembrolizumab.^[36]^ Given the discordant evidence, the utility of STK11 as a reliable predictive biomarker warrants further validation. In patients harboring KRAS mutations and complex comorbidities, the exigency for personalized therapeutic intervention is underscored, particularly given the circumscribed treatment options dictated by the presence of additional mutated genes.

#### 4.1.2. The tumor microenvironment

The tumor microenvironment (TME) encompasses a complex milieu of cellular and extracellular constituents, including but not limited to vascular structures, immune cells, fibroblasts, inflammatory mediators, and signaling molecules.^[37,38]^ These extracellular elements, nourished by the vasculature, consist of a range of cytokines, growth factors, hormones, and diverse stromal and immune cell populations, such as T and B lymphocytes.^[39]^ The burgeoning exploration of immunotherapeutic modalities, explicitly tailored to the unique characteristics of KRAS-induced TME, has surfaced as a pivotal area of academic inquiry and may amplify the efficacy of existing immunotherapy regimens.^[40,41]^ Antiangiogenic agents, representing a predominant class of therapeutics targeting the TME, function by inhibiting neovascularization essential for tumor proliferation and dissemination.^[42,43]^ A seminal study combined Ramucirumab with pembrolizumab across advanced gastric or gastroesophageal junction adenocarcinoma, non-small cell lung cancer, and uroepithelial cancers, revealing encouraging antitumor activity and a well-tolerated safety profile. This research buttressed the potential synergistic benefits of concomitant VEGF-VEGFR2 and PD-1-PD-L1 inhibition.^[44]^ Such integrative strategies, utilizing modulators of the TME in conjunction with immunotherapy, are under active clinical investigation for KRAS-mutated malignancies, with the promise of broadening the therapeutic armamentarium.^[45,46]^

In this discussion, we present a hypothesis. The effectiveness of immunotherapy in the context of KRAS mutations co-occurring with either STK11 or KEAP1 mutations presents a complex picture, characterized by varied outcomes across different clinical trial frameworks. It suggests the possibility that integrating antivascular therapeutic approaches might counteract the immunosuppressive effects associated with these co-mutations, thereby enhancing the treatment’s efficacy. However, the practicality of such an intervention necessitates thorough investigation. It is crucial to determine not only its efficacy but also its safety, especially in cases involving isolated KRAS mutations, before co-mutation studies can be confidently pursued.

### 4.2. Analysis of clinical trials

Research on clinical trials illustrates the focus of attention, summarizes the effects of drugs and combinations, and provides a quick overview of current research directions.

#### 4.2.1. Immune checkpoint blockades

Clinical evidence increasingly underscores the potential for immunotherapeutic strategies to benefit patients harboring KRAS mutations. The seminal Keynote-042 trial delineated that patients with KRAS mutations manifest superior response rates to ICB relative to their KRAS wild-type counterparts.^[47]^ Prior to the advent of immunotherapeutic modalities, the standard of care for KRAS mutations predominantly consisted of cytotoxic chemotherapy; thus, comparative analyses between chemotherapy and immunotherapy stand as the most robust testament to the latter’s therapeutic superiority.^[48,49]^ An open-label, phase 3 randomized controlled trial in patients with PD-L1-positive non-small cell lung cancer (NSCLC) revealed a median PFS of 10.3 months in the pembrolizumab arm compared to a mere 6.0 months in the chemotherapy arm, emphatically highlighting the therapeutic advantage of immunotherapy.^[17]^ Of note, patients in both comparative cohorts were PD-L1-positive. However, for PD-L1 expression-negative patients, ICBs still confer clinical benefit, albeit at a reduced efficacy as compared to their PD-L1-positive counterparts (negative: HR, 0.79; 95% CI, 0.67 to 0.93; weak-positive: HR, 0.80; 95% CI, 0.67 to 0.95; strong-positive: HR, 0.61; 95% CI, 0.47 to 0.78).^[50]^ In scenarios characterized by suboptimal clinical outcomes, the imperative for incorporating combination therapeutic regimens becomes increasingly salient.

#### 4.2.2. ICB combined with chemotherapy

The amalgamation of chemotherapy with immunotherapy has garnered attention for its potential to enhance therapeutic outcomes, possibly via the upregulation of PD-L1 expression engendered by adjuvant chemotherapy.^[51]^ A retrospective study comprising 95 patients with advanced KRAS-mutated NSCLC corroborated this notion. The data demonstrated that PFS for patients receiving first-line ICBs in conjunction with platinum-based chemotherapy was statistically superior to monotherapeutic strategies (7.4 months vs 4.5 months, *P* = .035). Moreover, PFS for second-line ICB monotherapy compared to single-agent chemotherapy also favored the former (4.8 vs 3.0 months, *P* = .043). Intriguingly, KRAS mutational status did not influence these findings (PFS 5.267 months vs 6.734 months, *P* = .969).^[52]^ It is plausible that the synergy between chemotherapy and immunotherapy masks the disparity in efficacy related to KRAS mutation status. Consistent with these observations, an FDA-supported study posits that the combination of chemotherapy and ICB is superior to either modality in isolation for NSCLC patients.^[53]^

Conversely, evidence gleaned from a randomized phase II trial indicated that the concomitant use of checkpoint inhibitors (durvalumab and tremelimumab) with chemotherapy agents (gemcitabine and nab-paclitaxel) did not confer a survival advantage in patients with metastatic pancreatic ductal adenocarcinoma (mPDAC) compared to chemotherapy alone.^[54]^ These findings raise the intriguing possibility that the anatomical origin of KRAS mutations may influence the therapeutic efficacy of combined modalities. However, the data should be interpreted cautiously given potential limitations such as insufficient sample size.

#### 4.2.3. ICB combined with targeted therapy

##### 4.2.3.1. ICB combined with KRAS-G12C inhibitor

The therapeutic milieu has been invigorated by the advent of KRAS-G12C inhibitors, namely sotorasib and adagrasib, in conjunction with ICB, most frequently pembrolizumab.^[55]^ Mechanistically, these combinations offer a compelling synergy; sotorasib selectively targets cysteine residues in KRAS proteins harboring the G12C mutation, thereby counteracting mechanisms of immune evasion.^[56]^ A clinical trial encompassing 58 patients with advanced solid tumors and KRAS-G12C mutations provides empirical substantiation for this strategic approach. Concomitant administration of corticosteroids ameliorated the most prevalent severe adverse events, culminating in a median OS of 15.7 months.^[57]^ While these data fortify the viability of sotorasib and immunotherapy for malignancies characterized by KRAS-G12C mutations, caution is requisite. Firstly, the prevalence of KRAS-G12C mutations in pancreatic and colorectal cancers is suboptimal, underscoring the imperative for the discovery and validation of alternative targeted agents. Secondly, the adverse reaction profile, experienced by approximately one-fourth of the participants, warrants meticulous scrutiny in future investigations.

##### 4.2.3.2. ICB combined with PARPi therapy

Poly(ADP-ribose) polymerase (PARP) constitutes a pivotal enzymatic entity implicated in cellular repair mechanisms following DNA damage.^[58]^ The cell cycle, even in neoplastic cells, is contingent upon efficacious DNA repair to circumvent apoptosis. Clinically available PARP inhibitors (PARPi), including Olaparib, Niraparib, Rucaparib, and Talazoparib, have been investigated extensively in the context of several malignancies but remain uncharted territory in the realm of KRAS-driven tumors. To address this lacuna, the Phase II PRODIGE-GERCOR study has been initiated, endeavoring to ascertain the efficacy of maintenance therapy incorporating olaparib or selumetinib concomitant with durvalumab, stratified by BRCAness and KRAS somatic status, in patients with metastatic pancreatic adenocarcinoma (EUCTR2019-004366-18-FR).

##### 4.2.3.3. ICB combined with MEK inhibitors

MEK inhibitors act as molecular agents that thwart cellular growth and proliferation by mitigating the enzymatic activity of MEK1 and MEK2.^[59]^ Given that the Raf-MEK-ERK signaling cascade represents a critical downstream pathway of KRAS, blockade of MEK emerges as a strategic therapeutic avenue for curtailing neoplastic expansion in KRAS-mutated tumors.^[60]^ In a rigorously designed clinical trial juxtaposing the efficacy of pembrolizumab, trametinib, and stereotactic body radiotherapy in pancreatic carcinomas harboring KRAS mutations, the median OS was reported to be 24.9 months, with a 95% CI ranging from 23.3 to 26.5 months. Contrastingly, the cohort treated with stereotactic body radiotherapy in combination with gemcitabine exhibited a median OS of 22.4 months, with a 95% CI spanning from 21.2 to 23.6 months.^[61]^

##### 4.2.3.4. ICB combined with KRAS-G12D inhibitors

At present, there are 2 ongoing Phase 1/2 clinical trials specifically aimed at evaluating agents targeting the KRAS-G12D mutation. Both studies are investigating the efficacy and safety of ELI-002, a novel therapeutic formulation comprising lipid-conjugated immunostimulatory oligonucleotides, denoted as Amph-CpG-7909, in conjunction with lipid-coupled peptide antigens, designated as amph-peptides. These trials, registered under the ClinicalTrials.gov identifiers NCT04853017 and NCT05726864, are yet to report their results. Hence, the evidence base for the therapeutic utility of this approach remains nascent and awaits further empirical substantiation.

#### 4.2.4. Adoptive cell transfer therapy

##### 4.2.4.1. T cell receptor-engineered T cells

The advent of T-cell receptor-engineered T cells as a therapeutic modality represents a significant yet challenging frontier in the treatment of KRAS-mutated tumors.^[62,63]^ This innovative technique involves the transduction of T lymphocytes with a T cell receptor (TCR) gene that binds selectively to specific tumor-associated antigens, thereby imbuing these lymphocytes with an enhanced capacity to target and eliminate cancer cells.^[64]^ A case report has illuminated the therapeutic potential of TCR-T in treating metastatic pancreatic cancer characterized by a KRAS-G12D mutation, demonstrating rapid tumor regression following treatment failure with other modalities.^[65]^ Nonetheless, the efficacy of TCR-T remains limited in the context of most solid tumors, necessitating further research to optimize its application in KRAS-mutated neoplasms.^[66]^ Notably, the “off-target, off-tumor” effects observed in some TCR-T clinical trials pose a significant concern, as they can lead to detrimental effects on healthy tissues.^[67]^

##### 4.2.4.2. Vaccine

Cancer vaccine therapy, a therapeutic modality with roots extending back to 1999, presents a promising avenue for improving survival outcomes in patients with KRAS-mutant tumors.^[68]^ Clinical trials have primarily explored 2 categories of vaccines: chimeric antigen receptor dendritic cell vaccines and KRAS peptide vaccines.^[69]^ In a longitudinal analysis of resected pancreatic cancer patients vaccinated with a mutant K-ras construct, the response rate among the 27 patients evaluated was 28%. Notably, the median survival for those who responded was an impressive 29 months, in stark contrast to the 5.22-month median survival across all patients. The 10-year survival rate was identical for both cohorts at 20%. Intriguingly, the 0-year survival rate in the vaccinated group was 87%, a staggering improvement over the zero survival rate in the contemporaneously treated, unvaccinated group.^[70]^

Emerging candidates, such as AlloStim Cell therapy, represent the frontier of vaccine development. This unique approach involves injecting allogeneic T-cells into patients, leading to the generation of “memory h1” cells that subsequently target tumor cells upon repeated injections. When AlloStim is administered in later stages, these memory cells, along with natural killer cells, are mobilized to assail tumor cells.

In summary, vaccine therapies remain a feasible and increasingly promising therapeutic strategy, especially when considering the integration of novel vaccine types with agents that mitigate immunosuppressive effects (NCT02380443).

## 5. Conclusions

This comprehensive review and bibliometric analysis have illuminated the rapidly evolving landscape of immunotherapy in the context of KRAS-mutant cancers. Through our meticulous examination of 1420 articles and 52 clinical trials, we have underscored the paramount importance of ICBs, especially when combined with other therapeutic modalities like chemotherapy, in enhancing treatment outcomes for this challenging cohort of cancers. Our findings suggest that ICBs, TCR-T, and cancer vaccines represent the forefront of innovative approaches capable of transforming the management of KRAS-mutant tumors.

The bibliometric analysis revealed a marked increase in research activity, particularly from the United States and China, emphasizing the global commitment to tackling KRAS-mutant cancers. Clinical trials have particularly highlighted the potential of ICBs and the emerging role of combination therapies in providing new hope for patients with these mutations. Moreover, our exploration into the TME has identified it as a critical factor influencing the efficacy of immunotherapeutic strategies, pointing to the necessity for future studies to further unravel the complex interactions within the TME that could unlock new therapeutic targets.

Despite the promising advancements, our review also identifies significant gaps in the current understanding and application of immunotherapy in KRAS-mutant cancers. Notably, the variability in response to ICBs based on different KRAS mutations and the interplay with other genomic alterations presents a complex challenge that requires further elucidation. Additionally, the lack of comprehensive studies exploring the long-term effectiveness and safety of emerging therapies underscores the need for sustained and focused research efforts.

Looking ahead, the field stands on the cusp of significant breakthroughs with the potential to revolutionize the treatment paradigm for KRAS-mutant cancers. Key to this evolution will be the development of more precise and personalized therapeutic strategies, informed by a deeper understanding of the genetic and immunological landscape of each tumor. The integration of innovative technologies, such as artificial intelligence and machine learning, could further enhance the predictive accuracy of treatment outcomes, enabling more tailored and effective interventions.

In conclusion, while significant strides have been made in the understanding and treatment of KRAS-mutant cancers, the journey towards optimal therapeutic strategies is far from complete. Our study calls for a multidisciplinary approach that combines cutting-edge research, clinical trials, and technological advancements to pave the way for more effective, personalized cancer care. Future endeavors should aim to bridge the existing gaps in knowledge, with a particular focus on unraveling the complexities of the TME and exploring novel combinations of therapeutic modalities to improve patient outcomes in the fight against KRAS-mutant cancers.

## Author contributions

**Conceptualization:** Hongyang Liu, Min Qiang.

**Data curation:** Hong Wang, Rui Guo.

**Formal analysis:** Rui Guo.

**Funding acquisition:** Hong Wang, Rui Guo.

**Investigation:** Rui Guo.

**Methodology:** Hongyang Liu.

**Resources:** Hongyang Liu, Yang Xing.

**Software:** Hongyang Liu, Min Qiang, Ying Zhang, Rui Guo.

**Visualization:** Hongyang Liu.

**Writing – original draft:** Hongyang Liu.

**Writing – review & editing:** Hongyang Liu, Min Qiang, Ying Zhang, Yang Xing, Rui Guo.
